# A cluster-randomized controlled trial evaluating the effect of culturally-appropriate hypertension education among Afro-Surinamese and Ghanaian patients in Dutch general practice: study protocol

**DOI:** 10.1186/1472-6963-9-193

**Published:** 2009-10-22

**Authors:** Joke A Haafkens, Erik JAJ Beune, Eric P Moll van Charante, Charles O Agyemang

**Affiliations:** 1 Department of General Practice, Amsterdam Medical Center, University of Amsterdam, The Netherlands; 2 Department of Social Medicine, Academic Medical Center, University of Amsterdam, The Netherlands

## Abstract

**Background:**

Individuals of African descent living in western countries have increased rates of hypertension and hypertension-related complications. Poor adherence to hypertension treatment (medication and lifestyle changes) has been identified as one of the most important modifiable causes for the observed disparities in hypertension related complications, with patient education being recommended to improve adherence. Despite evidence that culturally-appropriate patient education may improve the overall quality of care for ethnic minority patients, few studies have focused on how hypertensive individuals of African descent respond to this approach. This paper describes the design of a study that compares the effectiveness of culturally-appropriate hypertension education with that of the standard approach among Surinamese and Ghanaian hypertensive patients with an elevated blood pressure in Dutch primary care practices.

**Methods/Design:**

A cluster-randomized controlled trial will be conducted in four primary care practices in Amsterdam, all offering hypertension care according to Dutch clinical guidelines. After randomization, patients in the usual care sites (n = 2) will receive standard hypertension education. Patients in the intervention sites (n = 2) will receive three culturally-appropriate hypertension education sessions, culturally-specific educational materials and targeted lifestyle support. The primary outcome will be the proportion of patients with a reduction in systolic blood pressure ≥ 10 mmHg at eight months after the start of the trial. The secondary outcomes will be the proportion of patients with self-reported adherence to (i) medication and (ii) lifestyle recommendations at eight months after the start of the trial. The study will enrol 148 patients (74 per condition, 37 per site). Eligibility criteria for patients of either sex will be: current diagnosis of hypertension, self-identified Afro-Surinamese or Ghanaian, ≥ 20 years, and baseline blood pressure ≥ 140/90 mmHg. Primary and secondary outcomes will be measured at baseline and at 3 1/2, 6 1/2, and eight months. Other measurements will be performed at baseline and eight months.

**Discussion:**

The findings will provide new knowledge on how to improve blood pressure control and patient adherence in ethnic minority persons with a high risk of negative hypertension-related health outcomes.

**Trial registration:**

ISRCTN35675524

## Background

Hypertension (HTN) is a major risk factor for cardiovascular morbidity and mortality [1]. In western countries, ethnic minority populations of African descent are disproportionately affected by HTN and HTN-related cardiovascular morbidity and mortality [2-4]. This has also been observed among two major immigrant groups of African descent in the Netherlands: African-Surinamese from the former Dutch colony of Suriname (hereafter referred to as Surinamese) [5], and Ghanaians [6,7]. Poor adherence to prescribed medication and lifestyle changes such as weight control, regular physical activity, avoidance of tobacco, and a moderate intake of salt and alcohol, has been identified as one of the most important modifiable causes for the observed disparities in hypertension related complications [8,9]. Enhancing patient adherence to such therapeutic measures can be an important first step towards eliminating disparities [4,6,9-11].

In the Netherlands, general practitioners (GPs) play an important role in the treatment of HTN. European and Dutch primary care guidelines recommend patient education as a means of improving patients' motivation for and ability to adhere to HTN treatment goals [12,13]. Medical perceptions of disease and treatment can differ from lay perceptions [14], and patients can have a major impact on adherence to treatment [15-17]. HTN educators are therefore advised to employ "patient-centred" educational approaches that allow them to explore individual patients' beliefs and needs, and to find common ground regarding treatment [18]. The Dutch guidelines recommend focusing on the "5 As" (ask, assess, advise, assist and arrange) [19]. Studies from the UK and the USA have shown that patient beliefs about HTN and treatment can differ between ethnic groups [20-24]. This was also a finding from one of our previous studies, which focused on White Dutch, Surinamese and Ghanaian hypertensive patients living in the Netherlands [25]. Indeed, there is increasing evidence, mainly from diabetes research, that "culturally sensitive" patient education may have a positive influence on medication use and lifestyle changes among ethnic minority patients [26,27]. Culturally-appropriate patient education typically combines the principle of "patient-centred" care with that of "culturally competent" care [18]. To date, however, the European and Dutch HTN guidelines for GPs have not provided any recommendations on how HTN educators might address cultural variations in patients' perceptions of HTN. Moreover, the literature offers few descriptions or evaluations of means by which this might be achieved [11,28].

In a previous project, "Under Pressure 1", we developed a multi-component provider intervention to facilitate culturally-appropriate HTN education (CAHE) in Dutch primary care practices. This intervention mainly consisted of a CAHE toolkit that could be used to supplement the standard approach to HTN education recommended by Dutch clinical guidelines [1]. To support HTN educators using the toolkit, we developed an educational course and a framework for feedback meetings. A pilot undertaken in six primary care health centres (PCHC) in South East Amsterdam demonstrated that healthcare providers who had received the intervention thought it more important to take a patient's cultural background into account when delivering care. They also experienced fewer barriers to delivering culturally-appropriate health education to patients with cardiovascular problems than the control group (Beune EJAJ, Haafkens JA, Mohrs J, Stronks K, Bindels PJE: Pilot study evaluating the effects of an intervention to enhance culturally appropriate hypertension education among health care providers in a primary care setting, submitted). In the pilot practices, general practice assistants (GP assistants) and nurse practitioners (NP) were responsible for conducting HTN education under the supervision of GPs. A qualitative study of the experiences of the professionals who had piloted the intervention revealed a number of contextual barriers that had hampered the implementation of the CAHE toolkit in daily practice (Beune EJAJ, Haafkens JA, Bindels PJE: Pilot study of barriers and enablers influencing the implementation of an intervention to stimulate culturally appropriate hypertension education in a primary care setting, submitted). Frequently-mentioned problems included PCHCs being under-resourced, ongoing organizational changes, limited access to office space, the rapid turnover of GP assistants, and the fact that initially, GP assistants lacked the skills to conduct patient-centred HTN. Fortunately, many of these barriers could be addressed using well-known strategies for supporting innovations in primary care [29-31]. We therefore concluded that it would be feasible for Dutch primary care practices to adopt and implement the intervention.

In this paper, we describe the design of and rationale for our next project, "Under Pressure 2 (UP2)". The aim of UP2 is to test, using a cluster-randomized trial, the effectiveness of the previously-developed CAHE intervention among hypertensive Surinamese and Ghanaian patients. The latter should be receiving care in a Dutch primary care setting, and should have an elevated blood pressure (that is, greater than or equal to 140/90 mmHg). Our primary hypothesis is that a higher proportion of patients randomized to the CAHE intervention will have a reduction in systolic blood pressure (SBP) greater than or equal to 10 mmHg at eight months after the start of the intervention, as compared to those randomized to the usual care condition. Our secondary hypotheses are that a higher proportion of patients randomized to the intervention will be adherent to the prescribed (i) medication and (ii) lifestyle recommendations at eight months after the start of the intervention, as compared to those randomized to the usual care condition.

## Methods

### Study design

**Figure 1:**
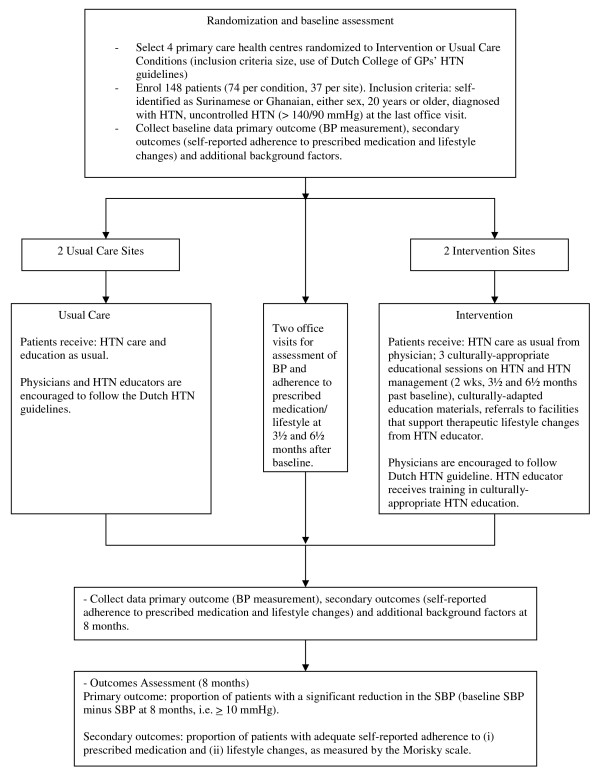
**Study design**.

The study will consist of a cluster-randomized controlled trial with two conditions: an intervention condition (IC) and a usual care condition (UC), as shown in Figure 1. Using a balanced design, four PCHCs will be randomly assigned to the IC (n = 2) or the UC (n = 2). Prior to randomization, the PCHCs will be matched on the size of the practices. A member of the Academic Medical Center's (AMC) Data Management Services team will carry out the randomization of the practices, and this team member will be blinded for the practices' identities. In those PCHCs assigned to the UC, the patients studied will receive the standard HTN education, based on the recommendations in the Dutch clinical guidelines [1]. In the PCHCs assigned to the IC, meanwhile, the patients studied will receive three CAHE sessions at two weeks post baseline, and three and six months thereafter. Data will be collected at baseline, during two office visits three-and-a-half and six-and-a-half months thereafter, and at eight months past baseline. The primary and secondary outcomes will be calculated based on data collected at baseline and eight months thereafter.

### Study sites

South East Amsterdam is one of the areas in the Netherlands that has a relatively high percentage of Surinamese and Ghanaian inhabitants. We have already selected four PCHCs in South East Amsterdam for the study. The inclusion criteria for each of the sites are that they (i) provide HTN care according to a practice protocol based on the Dutch clinical guidelines [1], and (ii) are not participating in similar studies to improve cardiovascular risk management.

### Study population

To be recruited for the study, patients have to fulfil the following eligibility criteria: (i) self-identified as Surinamese or Ghanaian; (ii) aged 20 or over; (iii) diagnosis of HTN with the International Classification of Primary care ICPC codes K85, K86 or K87; and (iv) an uncontrolled BP at the last office visit. Following the Dutch clinical guidelines, an elevated BP is defined as SBP/diastolic blood pressure (DBP) ≥ 140/90 mmHg. In addition, all patients must have an elevated BP at the baseline assessment meeting. Patients will be excluded if they have type 1 or type 2 diabetes, on the grounds that many diabetes patients with HTN may already be following diabetes education programmes that share characteristics with the intervention that will be offered in this trial. Patients will also be excluded if they are participating in other cardiovascular disease-related trials, if the GP who is treating them judges them to be unfit for participation (e.g. due to co-morbidity), or if they are unable or unwilling to provide informed consent.

### Sample size calculation

Our power analysis for sample size is based on the primary outcome measure of the study: SBP at baseline minus SBP at eight months after baseline. SBP has been selected because it is the most important factor for determining a patient's cardiovascular risk profile [1]. Furthermore, in almost all cases, the DBP will become lower if the SBP becomes lower. Following the literature, we consider a reduction of the SBP of 10 mmHg to be a clinically relevant difference [16,32].

The following rationale was used for the power calculation of the sample. The trial will be conducted in four PCHCs. We assume that patients in the IC and the UC will have similar demographic characteristics and that they will have been treated similarly, in line with the Dutch clinical guidelines [1]. We therefore expect there to be no differences between the four PCHCs with respect to the mean BP of patients with HTN. We can assume that the inter-cluster correlation is low (3%), because this is a ratio of the between-cluster and the low intra-cluster variance. In order to demonstrate a statistical difference of 10 mmHg with a standard deviation (SD) of 15 and a two-sided alpha of 5% and a power of 80%, we need a sample size of 148 patients (74 per treatment condition and 37 per PCHC). As we expect a drop-out rate of 40% prior to inclusion, we aim to recruit 246 patients.

Registry data from the four PCHCs that have been included in the study show that each PCHC has about 100 Surinamese and 38 Ghanaian registered patients who are being treated for HTN (K85,86, K87). Based on data from Agyemang et al. [5], it is estimated that about 63% of these patients will have a blood pressure > 140/90 mmHg. We therefore expect that we will be able to obtain an adequate sample size for this study.

### Approvals and data and safety monitoring

The study has been approved by the AMC's Medical Ethics Committee, which forms part of the University of Amsterdam in the Netherlands (protocol ID MEC09/070). Participants will provide written informed consent prior to enrolment. Recruitment procedures will be conducted in accordance with the Dutch Medical Research Involving Human Subjects Act and the World Medical Association Declaration of Helsinki.

### Patient recruitment

The following steps will be taken in order to recruit patients. First, the electronic medical records (EMR) from the four selected PCHCs will be reviewed in order to select all patients who meet the inclusion criteria for HTN, age and sex. As Dutch EMRs provide no information on patient ethnicity, attending physicians will help to identify Ghanaian and Surinamese patients according to their knowledge of the patients or their names. The relevant patients' GPs will be informed of their potential eligibility, and the GPs will be asked for their permission to enrol the patients in the study. Eligible patients will receive a letter from the researchers by post, which will have been co-signed by their GP. This letter, which will be written in Dutch (Surinamese patients) or English (Ghanaian patients), will inform patients about the study and invite them to participate. It will include a pre-paid postcard that can be sent back to the research staff if the patient concerned wishes to participate in the study. If a patient fails to react, a member of the research team will call him or her to offer more information about the study and discuss participation.

If a patient expresses interest in participating, a staff member will check whether they meet the eligibility criteria, and will ask whether they are willing to sign an informed consent form. Eligible patients who agree to sign this form will be invited to attend a one-hour baseline assessment meeting. After making sure that the patient has signed the consent form, a research assistant (RA) will use this meeting to assess a patient's SBP and DBP, height, weight, and BMI, using standardized techniques (see below), and to obtain self-reporting measures using the baseline questionnaire. Patients who do not meet the inclusion criteria for BP or ethnicity will not be included in the study. These patients will instead receive the standard five minutes of coaching about HTN care from the RA who conducts the baseline measurement. Patients from IC practices will be assigned to the IC condition, while those from UC practices will be assigned to the UC condition. The RA, who will be blinded for the IC of the patient's PCHC, will refer all eligible patients to their PCHC's GP assistant. The GP assistant will then provide patients with a letter outlining further steps in the study. They will also make appointments with patients for follow-up office visits, in line with instructions received from the research staff. To encourage participation, all patients who are included in the study will be promised a small reward or present (value of 25 euros) after they have completed the final assessments, regardless of the intervention site.

### Interventions

#### Intervention condition (IC)

Patients in the IC practices will receive HTN care as recommended in the Dutch clinical guidelines. Instead of the standard counselling to facilitate adherence to medication and therapeutic lifestyle changes, patients will receive: (i) three culturally-appropriate HTN education (CAHE) sessions over a period of six and a half months, conducted by a trained NP; (ii) culturally-specific, educational written material; and (iii) if necessary, referrals to neighbourhood facilities that may help Surinamese and Ghanaian patients to adopt healthier lifestyles. Moreover, (iv) prior to the second and third counselling session, an assessment of each patient's BP and self-reported medication and lifestyle adherence will be made using standardized measures. These measures are described below.

#### Culturally-appropriate HTN education (CAHE)

The first session will take place two weeks after the baseline assessment interview, and the next two sessions will occur three and six months thereafter. The Dutch clinical guidelines recommend the 5 As [19] as the preferred strategy for supporting patients in achieving treatment goals, such as adherence to prescribed medication, dietary changes, weight loss, reduced sodium intake, increased physical activity and moderate use of alcohol [1]. While CAHE also uses this framework, it has the additional aim of eliciting and discussing culturally-specific aspects of patients' perceptions of HTN and HTN treatment. This method is based on the work of Arthur Kleinman [33], as well as more recent approaches to improving adherence in patients with HTN [34] that were further adapted and piloted in our previous study. In short, after identifying potential communication barriers and establishing a rapport with the patient, the first session will focus on the patient's beliefs about HTN. The next two sessions will deal with the daily challenges presented by achieving HTN treatment goals within the broader context of the patient's life (see Additional file 1 and 2).

#### Provision of information

Patients will also be given information leaflets that provide answers to frequently-asked questions about HTN. These will be designed to address the specific languages, customs, habits, norms and dietary cultures that characterize the Surinamese and Ghanaian communities.

#### Supporting healthier lifestyles

If necessary, patients will be referred to neighbourhood facilities offering healthier lifestyle support that is tailored to Surinamese and Ghanaian patients, based on a referral list that will be established for this purpose.

#### The counsellor

In order to ensure treatment fidelity and to avoid previously identified organizational- and healthcare-related obstacles to implementation, we will appoint one NP who will provide CAHE to all IC patients. The NP will have extensive training and experience in using the counselling approach recommended in the Dutch clinical guidelines (the 5 As). Prior to the intervention, she will receive additional training in order to improve her knowledge of HTN in the Ghanaian and Surinamese communities, general cross-cultural counselling techniques, and the specific CAHE method that will be used in this study. The training manual and methods that were developed during our previous study will be used for this purpose. During the trial, the NP will also be offered an opportunity to receive feedback from a member of the research team.

### UC condition

Patients in UC sites will receive standard HTN care and education, following the recommendations in the Dutch clinical guidelines [1]. After completing the baseline assessment, each patient will be given appointments for two office visits at three-and-a-half and six-and-a-half months thereafter for the assessment of their BP and self-reported adherence to medication and lifestyle changes.

Regardless of the randomization condition, it will be possible to charge all office visits carried out in the context of this trial to health insurance companies.

### Outcomes, measures and data analysis

#### Primary outcome

The primary outcome measure is the proportion of patients with a significant reduction in the SBP (10 mmHg; SD = 15) at eight months after inclusion.

#### Secondary outcomes

The secondary outcome measures are: (i) the proportion of patients with adequate adherence to prescribed medication at eight months after inclusion; and (ii) the proportion of patients with adequate adherence to lifestyle recommendations at eight months after inclusion.

Data will also be collected with respect to factors that characterize the patient group (baseline demographics, baseline medical chart data) and a number of factors that may influence patients' HTN management (perceptions of HTN, perceptions of medications, self-efficacy, experience of social support in HTN management, and level of satisfaction with care). In addition, data relating to the process of implementing the intervention will be collected.

#### Measures

The measurements to be studied can be divided into five categories: (1) physiological measures, (2) self-reporting measures, (3) pharmacy data, (4) chart data and (5) process data. Table 1 summarizes the measures according to the timeline. A trained RA will conduct all physiological and self-reporting measures at baseline and at eight months. Attending NPs at the IC and UC practices will take a subsection of these measures (BP and self-reported adherence to medication and life style changes) at three-and-a-half and at six-and-a-half months. A member of the research staff will collect pharmacy and chart data. Time registration and patient drop-out data for the process analysis will be collected using registrations made by the RA and the NPs. A member of the research staff will conduct post-intervention interviews with patients.

**Table 1:** Measures used in the trial and timeline

**Measures**	**Baseline**	**2** **wks**	**3 1/2** **mnths**	**6 1/2** **mnths**	**8** **mnths**	**9** **mnths**
**Physiological measures**						
- Office BP measurements	X		X	X	X	
- Height, weight, BMI	X				X	
- Hip and waist size	X				X	
**Self-reporting measures**						
- Patient demographics	X					
- Additional cardiovascular risk factors (physical activity, smoking, alcohol, sodium intake)	X				X	
- Medication adherence	X		X	X	X	
- Adherence to lifestyle recommendations	X		X	X	X	
- Perceptions of HTN	X				X	
- Perceptions of medication	X				X	
- Self efficacy	X				X	
- Experience of social support in HTN management	X				X	
- Satisfaction with care	X				X	
- Discrimination	X				X	
- Perceived stress	X				X	
**Pharmacy data**						
- Refill dates	X		X	X	X	
**Chart data**						
- Prescribed medication	X					
- Prescribed lifestyle measures	X					
- Co-morbidity	X					
**Process data**						
- Registration office visits, patient drop-out	X	X	X	X	X	
- Patients interviews						X

#### Physiological measures

BP will be measured three times using an automated BP monitor (Omron 705-IT), with the patient seated comfortably for five minutes. The average of the last two readings will be used as a measure for each visit. The same procedure will be used by trained NPs during office visits at three-and-a-half and six-and-a-half months after baseline.

Height and weight will be measured in the absence of shoes and heavy clothing, using a tape rule and a validated scale respectively. All measurements will be recorded to the nearest 0.1 cm and 0.1 kg. These measures will be used to compute a patient's BMI. Waist size will be measured by putting the tape horizontally around the smallest part of the waist. Hip size will be measured by putting the tape horizontally around the widest part of the hips.

#### Self-reporting measures

In order to collect socio-demographic data describing the study population, we have adapted an instrument used in an earlier study [35]. The variables will include age, gender, self-identified ethnicity, educational level, employment status, household income, marital status, household composition, duration of stay in the Netherlands, duration of HTN, and health insurance status.

To establish the presence of additional cardiovascular risk factors, we will use an instrument that contains six questions relating to physical activity, smoking, alcohol and sodium intake. Questions will be based on an instrument used in a previous study [35].

Adherence to prescribed antihypertensive medication will be measured with the widely-used four-item scale developed by Morisky [36]. This instrument has been well validated in studies of African American inner-city populations [37-39]. The scale asks patients to respond with "yes" or "no" to a set of four questions. A positive response to any question indicates a problem with adherence. Each positive answer is assigned a score of 1. The total possible score is 4, with a higher score indicating a poorer level of adherence. In this study, patients with a score of ≥ 1 will be categorized as non-adherent. In addition, six items will be used that are based on our previous study [40].

To assess adherence to lifestyle recommendations, we will use four items derived from the Morisky scale [36] adapted for the purpose of this study.

Perceptions of HTN and HTN treatment will be measured using 52 items: of these, nine will be from the IPQ-brief [41], 18 will be from the IPQ-R HTN [42], five will be derived from our earlier studies on perceptions of HTN and HTN management among Surinamese and Ghanaians [40,43,44], and 20 items will be adapted from the IPQ-R [45]. The validity and the reliability of the IPQ-brief and the IPQ-R are well established [41,45,46]. Patients' perceptions of antihypertensive medications will be measured using the ten-item BMQ [47], an instrument that has good internal consistency and validity [48,49]. In addition to this, we will add three items that are based on our previous study [40].

To measure patients' self-efficacy in HTN management, the 13-item MASES-R will be used. This instrument has good internal consistency and predictive and convergent validity [50].

Social support will be measured using the 12-item DUSOCS [51], which, as demonstrated by previous studies, has good validity and reliability [37].

Satisfaction with care will be measured using six items of the Consumer Quality Index-diabetes [52]. Similar Consumer Quality Index instruments have demonstrated good construct and discriminating validity and internal consistency in previous Dutch studies [53,54]. In addition, five items from the Quote-Migrant will be used, which has been specifically developed to measure the experienced quality of care in migrant populations [55].

Socio-demographic data will only be measured at baseline. Most of the other self-reporting measures will be collected at baseline and at eight months thereafter.

The Morisky scale will also be used to assess adherence to medication and lifestyle recommendations during the second and third office visits, respectively three-and-a-half and six-and-a-half months after the baseline assessment. Patients will be asked to complete these questionnaires in practice waiting rooms, prior to meeting the NP.

Discrimination and stress will be measured with two scales from the CAATCH trial [56].

#### Pharmacy data

In addition to self-reporting data, pharmacy data will be used to check self-reported adherence to medication. These data will allow us to measure refill adherence; that is, the interval between the day when a prescription ends, and the day on which a new prescription is refilled [57]. A patient is defined as adherent if the prescription is refilled within seven days of the designated refill date. This method has also been used in other studies [58].

#### Chart data

In addition to self-reported biographical and disease data, relevant chart data from the patients' charts will be extracted from the EMR as soon as patients have been included in the study. The following data will be extracted: SBP and DBP measured during the last two office visits; prescribed medication; the presence of additional risk factors for cardiovascular disease (physical exercise, diet, sodium, smoking, alcohol, height, weight, BMI, waist and hip circumference, glucose, cholesterol); and the presence of previous cardiovascular diseases (myocardial infarction, decompenstatio cordis, TIA, CVA), kidney insufficiency, liver disease, dementia or mental health problems. In a previous study, we found that GP registration data can sometimes be incomplete [40]. If these data are indeed sufficient, they will be used as additional information to describe the background characteristics of the study population, and to control for confounders.

#### Process data

Time registrations for office visits and additional activities made by the RA and NPs will be used to make a cost analysis of the intervention. Registration data on patient drop-out will be used to analyse the feasibility of undertaking the intervention for the patients groups in question. Moreover, qualitative interviews with patients in the IC and the UC conditions are planned to explore patients' experiences of barriers to and incentives for participating in the study. We will also enquire about non-reimbursable expenses that might arise from participating in the study, such as travel costs and costs related to lifestyle changes.

#### Blinding

Due to the nature of the interventions concerned, complete blinding of investigators and professionals working in the PCHCs would be impossible. However, the RA who will conduct the assessments at baseline and at eight months will be masked for the IC of the PCHCs. All BP measurements will be taken with a standard automated measurement device that can prevent ascertainment bias. Even though physicians may be aware of the IC of their healthcare centre, we consider contamination to be unlikely on the grounds that the intervention (CAHE) will require the use of specific expertise and tools. Finally, data managers will be strictly blinded for the IC. All preliminary data will be stored in one dataset until the end of the intervention.

#### Planned data analysis

All imported data will be controlled at random for miscodings. As a first step, univariate analyses will be performed to compare the distribution of variables and to identify abnormalities.

Primary and secondary outcomes will be calculated according to the "intention to treat" principle. The primary outcome measure, which will be calculated for every patient, will be the observed baseline SBP minus the SBP at eight months. Subsequently, the mean within group difference will be calculated separately for patients in the IC and UC conditions. Finally, the difference between these within group differences will be calculated with the accompanying SD. As stated previously, we regard a reduction of the SBP of 10 mmHg with a SD of 15 and a two-sided alpha of 5% in the IC group (as compared to the UC group) as a clinically relevant difference.

The secondary outcomes will be calculated using the dichotomous outcomes of measurements on the Morisky scale with respect to adherence to prescribed medication and lifestyle changes. The analytical procedure will be similar to that described for the primary outcome measure.

In a further analysis of the primary and secondary outcomes, we will use univariate and multi-level analyses that will include other data that may potentially affect the outcomes. If the N is sufficient, we will perform separate subgroup analyses for gender and ethnicity. A p of 0.05 will be used as the critical value for all analyses. For subgroup analyses, the p value will be corrected according to the number of analyses conducted.

## Discussion

In western countries, there is an urgent need to improve BP control among hypertensive patients of African descent. In the US, trials of interventions to enhance BP control and patient adherence to prescribed medications and lifestyle changes have focused on African American patients. Two of these trials are currently taking place [11,56] and one recently finished [37]. To our knowledge, the present study is one of the first European trials to address this issue. The US trials are evaluating the effect of interventions that are based on the principle of motivational interviewing [59]. The specific contribution of the present study is that the intervention builds on the principles of "culturally-appropriate counselling" [60], which can be used to supplement motivational interviewing. Another key characteristic of the intervention is that it has been developed and piloted using data from patients' own perceptions of HTN [40,43,44]. Moreover, drawing on theories of effective quality improvement in primary care [30,31], the feasibility for implementation by care providers has already been established in a previous pilot study.

If the results are positive, we hope that this intervention will find wider application, so that it may contribute to efforts to reduce ethnic disparities in HTN outcomes.

## Abbreviations

(AMC): Academic Medical Center; (CAHE): Culturally-appropriate hypertension education; (DBP): Diastolic blood pressure; (EMR): Electronic medical record; (GP): General practitioner; (GP assistant): General practice assistant; (HTN): Hypertension; (IC): Intervention condition; (NP): Nurse practitioner; (PCHC): Primary care health center; (RA): Research assistant; (SD): Standard deviation; (SBP): Systolic blood pressure; (UP2): Under Pressure 2; (UC): Usual care condition; 5 As: (ask, advise, assess, assist, arrange).

## Competing interests

The authors declare that they have no competing interests.

## Authors' contributions

JH wrote the research proposal for the funding organization, and drafted the manuscript of this paper. EB made a major contribution to the design of the research proposal, prepared the study protocol for ethical approval together with JH, and helped to draft this paper. CA participated in the design of the research proposal and the final drafts of the study protocol. EM commented on the final draft of the study protocol. All four authors read and approved the final manuscript of the paper.

## Pre-publication history

The pre-publication history for this paper can be accessed here:



## Supplementary Material

Additional file 1 **Summary of three culturally-appropriate hypertension education sessions**. The information provided describes the content of culturally-appropriate hypertension education. Click here for file

Additional file 2 **Topic list for eliciting a patient's explanatory model of hypertension**. The information provided describes the content of culturally-appropriate hypertension education. Click here for file
