# Supplementary material for: A cluster-randomized controlled trial evaluating the effect of culturally-appropriate hypertension education among Afro-Surinamese and Ghanaian patients in Dutch general practice: study protocol
Source: BMC Health Serv Res. 2009 Oct 22;9:193. doi: 10.1186/1472-6963-9-193 (PMC2771011; doi:10.1186/1472-6963-9-193)
Supplement: Additional file 1 — Summary of three culturally-appropriate hypertension education sessions. The information provided describes the content of culturally-appropriate hypertension education. [file 1472-6963-9-193-S1.DOC]

**Additional file 1.**

**Summary of three culturally-appropriate hypertension education sessions**

| Sessions | **Content sessions** | | | |
| --- | --- | --- | --- | --- |
| Main topic | **Method and topics to be addressed** | | | |
|  | **Elicit patient-perspective, using framework for culturally-sensitive communication** | **Inform patient about medical perspective** | **Reach consensus** | **Establish** |
| *Session 1*  1. Establishing communication barriers and rapport  2. What is hypertension? | - Experience of (culturally specific) communication barriers (i)  - What is hypertension? (i)  - Hypertension treatment and goals? (i) | Discuss  - Hypertension (ii)  - Treatment goals (ii) | - What is hypertension? (iii)  - Patient’s treatment goals? (iii) | - Potential barriers/facilitators for achieving treatment goals (i, iii)  - Goal for next 3 months |
| *Session 2 and 3*  1. How to achieve hypertension treatment goals? | Experience of (culturally specific) barriers/enablers in achieving patient’s hypertension treatment goals: medication use and lifestyle changes (i) | Discuss patient’s current: (i, ii, iii)  - BP measurement  - Self-reported medication and lifestyle adherence    - Treatment goals | - What feasible steps are needed to maintain/achieve treatment goals? (iii) | - Potential barriers/facilitators for achieving treatment goals (i, iii)  - Goal for next 3 months |

(i) Using culturally-sensitive counselling technique (Additional file 2); (ii) using information from hypertension guidelines [1,13]; (iii) using “5 As” as method [19].
