# Supplementary material for: A cluster-randomized controlled trial evaluating the effect of culturally-appropriate hypertension education among Afro-Surinamese and Ghanaian patients in Dutch general practice: study protocol
Source: BMC Health Serv Res. 2009 Oct 22;9:193. doi: 10.1186/1472-6963-9-193 (PMC2771011; doi:10.1186/1472-6963-9-193)
Supplement: Additional file 2 — Topic list for eliciting a patient's explanatory model of hypertension. The information provided describes the content of culturally-appropriate hypertension education. [file 1472-6963-9-193-S2.DOC]

**Additional file 2.**

**Topic list for eliciting a patient’s explanatory model of hypertension^1^**

| **Communication**   - Determine how a patient wants to be addressed (formally or informally) - Determine the patient’s preferred language for speaking and reading (Dutch or another language) - Use this information in your interaction with the patient |
| --- |
| **Introduction**   - It is often difficult for us (care providers) to give advice about hypertension and how to manage it if we are unfamiliar with our patients’ views and experiences. For this reason I would like to ask you some questions to learn more about your own views on hypertension and its treatment. |
| **Elicit personal views on hypertension and its treatment** |
| **Understanding**   - What do you understand hypertension to mean?   **Causes**   - What do you think has caused your hypertension? Why has it occurred now/when it did; why to you?   **Meaning and symptoms**   - What does it mean to you to have hypertension? - Do you notice anything about your hypertension? How do you react in this case?   **Duration and consequences**   - How do you think your hypertension will develop further? How severe is it? - What consequences do you think your hypertension may have for you (physical, psychological, social)?   **Treatment**   - What types of treatment do you think would be useful? - What does the prescribed therapeutic measurement(s) mean to you? |
| **Elicit contextual influences on hypertension management** |
| **Social**   - Do you speak with family/community members about your hypertension? How do they react? - Do family/community members help you or make it difficult for you to manage hypertension? Please explain. |
| **Culture/Religion**   - Are there any cultural issues/religious issues that may help you or make it difficult for you to manage hypertension? Please explain. |
| **Migration**   - Are any issues related to your position as an immigrant making it difficult for you to manage hypertension? Please explain. |
| **Finance**   - Are any issues related to your financial situation making it difficult for you to manage hypertension? Please explain. |

^1^Based on Kleinman’s Explanatory Model format [14,33] and our previous study [40,43,44].
